# A New Species of *Stigmatodactylus* (Orchidaceae) from West Papua Province, Indonesia, and the Rediscovery and Reinterpretation of *S. gibbsiae*

**DOI:** 10.3390/plants15040589

**Published:** 2026-02-13

**Authors:** André Schuiteman, Reza Saputra, Jimmy F. Wanma, Charlie D. Heatubun

**Affiliations:** 1 Royal Botanic Gardens, Kew, Richmond TW9 3AE, UK; charlie_deheatboen@yahoo.com; 2 Southwest Papua Natural Resources Conservation Agency, Ministry of Forestry, Jalan Klamono KM 16, Sorong 98418, Southwest Papua, Indonesia; reza.saputra@my.jcu.edu.au; 3 Tropical Biology and Conservation, College of Science and Engineering, James Cook University, McGregor Road, Smithfield, Cairns, QLD 4878, Australia; 4 Fakultas Kehutanan, Universitas Papua, Jalan Gunung Salju, Amban, Manokwari 98314, West Papua, Indonesia; jimmywanma@yahoo.com; 5 Program Studi S3 Biologi, Fakultas Matematika dan Ilmu Pengetahuan Alam, Universitas Indonesia, Jalan Margonda Raya, Depok 16424, West Java, Indonesia; 6 Badan Riset dan Inovasi Daerah (BRIDA) Provinsi Papua Barat, Jl. Brig. Jend. Mar. (Purn.) Abraham O. Atururi, Kompleks Perkantoran Gubernur, Arfai, Manokwari 98315, West Papua, Indonesia

**Keywords:** *Acianthus*, Arfak mountains, identification key, taxonomy

## Abstract

Two species of *Stigmatodactylus* have recently been observed in the Arfak Mountains of West Papua Province, Indonesian New Guinea. One of these is clearly undescribed and is here described as *Stigmatodactylus antennatus* Schuit., Saputra & Wanma, a species uniquely characterised by the presence of a pair of antennae on the lip. The other is believed to be *S. gibbsiae* (Kores) Kores. This species was only known from the type material collected in the Arfak Mountains more than a century ago, in 1913. There are, however, significant discrepancies between the protologue and the material recently found. We interpret these discrepancies as misinterpretations of the poor type material of *S. gibbsiae*, and provide images based on fresh material. An identification key to the four species of *Stigmatodactylus* in New Guinea is included.

## 1. Introduction

*Stigmatodactylus* Maxim. ex Makino (syn. *Pantlingia* Prain) is a genus of delicate and inconspicuous terrestrial orchids that inhabit the forest floor or sometimes grow on decaying logs [1,2]. It is distributed from North-East India and Bhutan to Japan in the North, and to the Solomon Islands and New Caledonia in the South. Currently, 29 species are recognised [3], plus *S. maraiparaiensis* S.F.Md.-Isa & A.S.Rob. The genus is morphologically similar to *Acianthus* R.Br., from which it was distinguished by Kores [4] on anatomical details. More recently, Clements & Jones [5] transferred several species that Kores had included in *Acianthus* to *Stigmatodactylus*, partly based on unpublished molecular studies by Lyon [6]. This implies that there is now a different set of characters distinguishing *Acianthus* from *Stigmatodactylus*. One of these is the presence of two viscidia in the pollinarium of *Acianthus* and one viscidium in *Stigmatodactylus*. The genus owes its name to an appendage (*dactylus*: finger) just below the stigma on the ventral side of the column. However, this appendage is indistinct or absent in some species; therefore, only its presence but not its absence is diagnostic.

In New Guinea, *Stigmatodactylus* was first recorded by Smith [7], who wrote: “Only in fruit but apparently belonging to this genus.” The specimens this referred to were later used by Kores to describe *S. gibbsiae* (Kores) Kores, initially in the genus *Acianthus* [8]. Kores simultaneously described two other species of *Acianthus* from New Guinea, which he transferred to *Stigmatodactylus* the following year [9]. These three comprise the hitherto known species of *Stigmatodactylus* in New Guinea.

During recent fieldwork in the Arfak Mountains of West Papua Province, Indonesia, a species of *Stigmatodactylus* was found that was obviously distinct from any known species of the genus by the presence of two slender antennae on either side of the labellar callus. This is here described as a new species.

In 2025, another species of *Stigmatodactylus* was found in the same area. Comparison with existing descriptions suggests that this material represents the first recollection of *S. gibbsiae* since its original discovery more than a century ago. However, several morphological features of the newly collected plants differ from those described in the protologue. We interpret these discrepancies as artefacts arising from the poor condition of the original type material, rather than evidence of taxonomic distinction.

## 2. Results and Discussion

**Figure 1:**
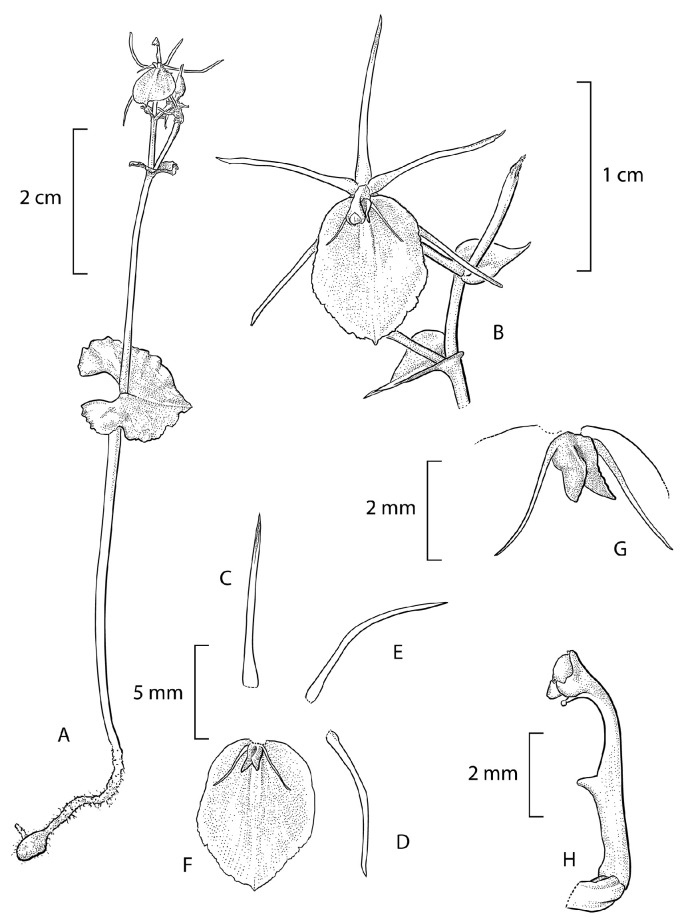
*Stigmatodactylus antennatus.* (**A**) Plant habit; (**B**) flower; (**C**) dorsal sepal; (**D**) lateral sepal; (**E**) petal; (**F**) lip; (**G**) lip callus; (**H**) column. Drawn after the type material by André Schuiteman.

**Figure 2:**
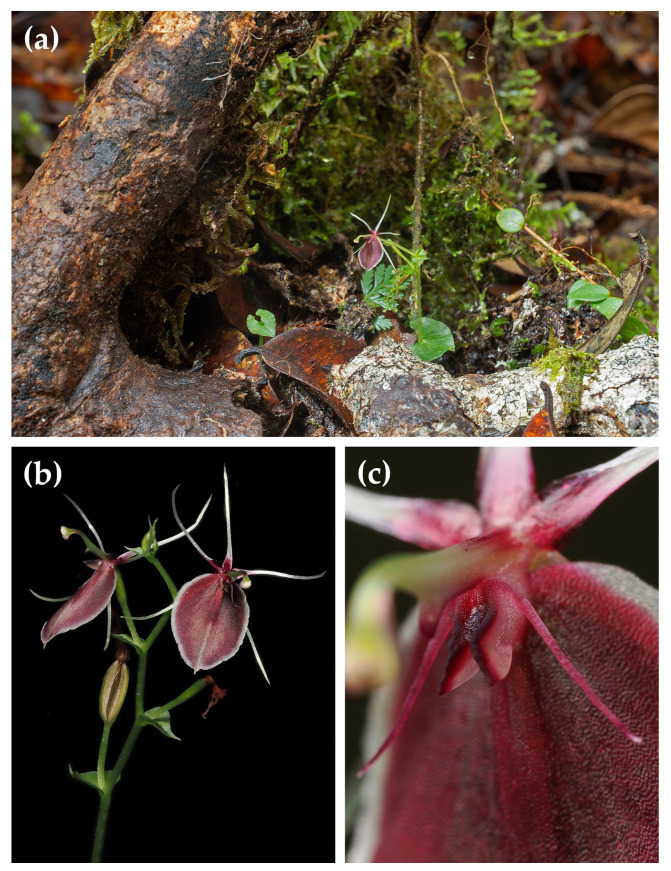
*Stigmatodactylus antennatus*. (**a**) In situ, *Schuiteman et al. 23-277*; (**b**) inflorescence, *Schuiteman et al. 24-102*; (**c**) flower detail showing callus with antennae, *Schuiteman et al. 23-277*. Photos: André Schuiteman.

**1. *****Stigmatodactylus antennatus*** Schuit., Saputra & Wanma, *spec. nov.* Type: Indonesia, West Papua Province, Arfak Mountains, Testega District, 17 October 2023, *A. Schuiteman*, *R. Saputra*, *A. Mofu*, *I. Farwas*, *C. Yokbari*, *and L. Jennings 23-277* (holotype MAN; isotypes BO, K). Figure 1 and Figure 2.

Diagnosis: Differs from all known species of *Stigmatodactylus* in the 2-winged apex of the labellar callus and in the presence of a long, slender antenna on either side of the callus.

Description: Small, tuberous herb. Tuber oblongoid, c. 3–5 mm long. Roots few. Roots and tuber covered with long, partly branching trichomes. Sometimes with a stolon forming at the base of the inflorescence on the adaxial side of the leaf. Stem 1-leaved, 5–6 cm long from apex of tuber to base of leaf, 1.3 mm diam., the basal c. 2 cm covered with scattered and partly branching trichomes, possibly functioning like roots, otherwise glabrous and without scale leaves or articulations. Leaf bright green, horizontally spreading, cordate to reniform, with rounded basal lobes, 1.1–1.6 × 0.6–2.4 cm, margins undulate, apex truncate to abruptly acute-acuminate; veins anastomosing at their apex just below the leaf margin. Inflorescence erect, 3–6.7 cm long, glabrous; peduncle not articulated, without peduncle-scales, 2–3 cm long; rachis laxly 3–6-flowered, glabrous, zig-zag, 1–3.7 cm long. Floral bracts foliaceous, green, broadly ovate to semi-orbicular, acute-acuminate, decreasing in size from the lowest flower upwards, the lowest bract c. 3–6 × 6.5 mm. Pedicel-with-ovary green, terete, slightly clavate, glabrous, 7.9 mm long. Flowers opening widely, one or two at a time; sepals and petals whitish-hyaline, reddish maroon at the very base, lip reddish maroon with a whitish-hyaline margin, callus pinkish white, the edges deep maroon; column greenish yellow, apical wings edged purple; anther cream colour. Sepals and petals glabrous, arranged in a 5-pointed star. Dorsal sepal and lateral sepals similar, narrowly linear, slightly concave lengthwise, with incurved margins, 9 × 0.3 mm in natural position, subcaudate-acuminate. Petals similar to the sepals but slightly wider, with incurved margins, 9.4 × 0.6 mm in natural position, sometimes with one or two small teeth in the apical part. Lip elliptic-ovate, entire, 8 × 6.2 mm, margins minutely ciliate, in apical half irregularly shallowly dentate, apex acute, mucronate, basal callus cup-shaped, adnate to the lip, 0.9 × 0.3 mm, 1.2 mm high in lateral view, just above the base, laterally on either side, with a very slender, linear-subulate, outwards-curving antenna 2.8 mm long, in front with a pair of vertical, laterally flattened, triangular, divergent wings 0.7 mm long, upper margin of the wings minutely crenulate. Column slender, erect, incurved at the apex, bilaterally flattened, 5.5 mm long; at 2.5 mm above the base just below the stigma with a porrect, triangular-digitate, 0.5 mm long appendage; at the apex with two semi-orbicular, downwards-pointing, 0.6 mm long wings; rostellum beak-like, 0.5 mm long, terminating in a semi-liquid, subspherical viscidium. Anther cucullate, suborbicular, 0.7 mm long and wide. Pollinia not seen. Fruit pedicellate, as long as the pedicel, oblongoid, c. 5.5 mm long, sharply 6-ribbed.

**Additional material studied:** Indonesia, West Papua Province, Arfak Mountains, Testega District, 9 November 2024, *A. Schuiteman, R. Saputra, J. Wanma et al. (leg. A. Trias-Blasi) 24-102*(MAN).

**Distribution:** Indonesian New Guinea, Bird’s Head Peninsula, Arfak Mountains, endemic.

**Habitat and Ecology:** Terrestrial between moss and leaf litter in fairly open montane forest. Elevation 1200–1250 m. Flowering October to November.

**Figure 3:**
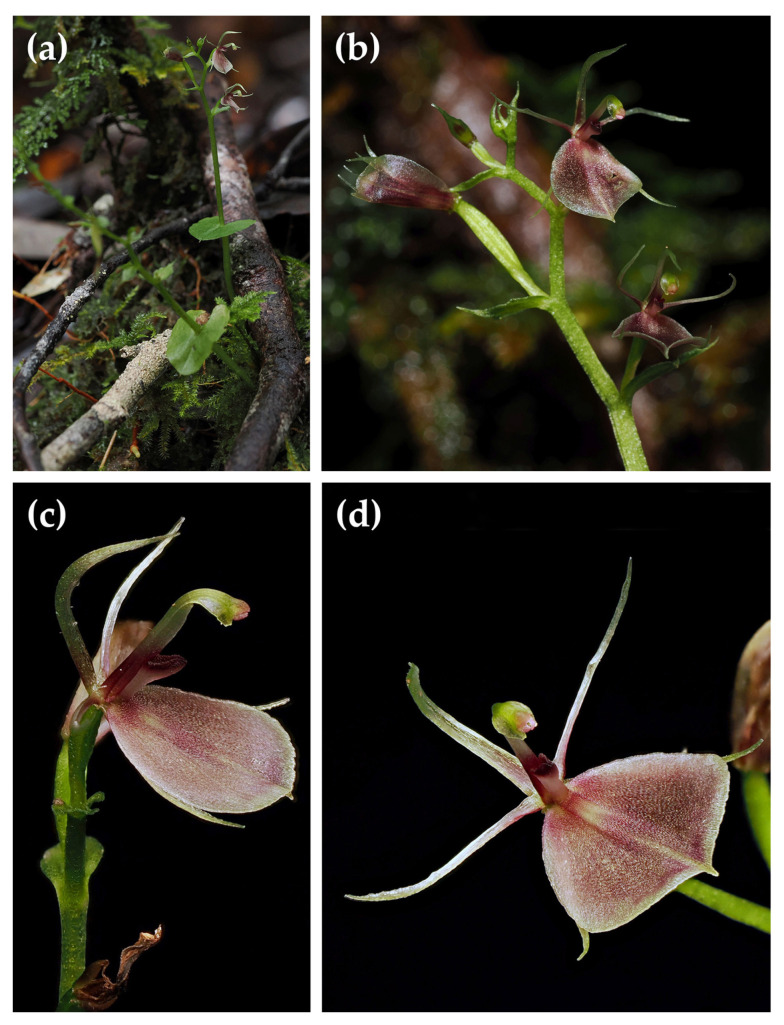
*Stigmatodactylus gibbsiae*. (**a**) in situ; (**b**) inflorescence; (**c**,**d**) flower, all from *Saputra et al. 301*. Photos: Reza Saputra.

**2.*****Stigmatodactylus gibbsiae*** (Kores) Kores, Novon 2(3): 212 (1992) (as ‘*gibbsae*’). *Acianthus gibbsiae* Kores, *Lindleyana* 6: 166 (1991) (as ‘*gibbsae*’). *Pantlingia gibbsiae* (Kores) Schuit., *Blumea* 39: 236 (1994). Type: Indonesia, West Papua Province, Arfak Mountains, Anggi Gida Lake (“Woman Lake” in [10]), c. 2100 m, December 1913, *L. S. Gibbs 5891* (holotype BM!). Figure 3.

**Additional material studied:** Indonesia, West Papua Province, Arfak Mountains, Testega District, c. 1080 m, 9 June 2025, *R. Saputra, A. Mofu, and Zainal 301 *(MAN).

**Distribution:** Indonesian New Guinea, Bird’s Head Peninsula, Arfak Mountains, endemic.

**Habitat and Ecology:** Terrestrial in montane forest. Elevation 1080–2100 m. Flowering June and December.

**Notes:** The type material of *Stigmatodactylus gibbsiae*, preserved at the Natural History Museum in London (BM), is poor, as the inflorescences either carry small flower buds or fruits. Only a few sepals or petals and a more-or-less damaged column are still attached to the fruits. There is no labellum visible. This material was deemed insufficient for description by J. J. Smith [7], who added the note “fruits only” to the specimen label, but was nevertheless used by Kores [8] to produce a detailed description and a line drawing. The collector, Lilian Gibbs, wrote: “On the north-east side [of Anggi Gida Lake], creeping under the bracken, the fine *Pterostylis papuana* var. *arfakensis*, from cream to brown-pink in colour, *Liparis lacus* a small plant with brown labellum and green petals, and a minute brown *Stigmatodactylus* sp. past flowering, grew on the forest edge” [9]. Since the flower colour could still be recorded, it appears that the flowers had withered but were not entirely desiccated.

The material that Kores dissected could not be found, except for what appeared to be a fruit with attached column. This was reduced to a transparent film sticking to a piece of paper, and one of us (AS) was unable to discern any morphological details on it. It is possible that Kores used ammonia to soften and reconstitute the material that he analysed, as he describes this method in one of his papers [4]. Unfortunately, material softened in this way must be stored in alcohol. It cannot be dried again, as most of the tissue will have been dissolved. We suspect that the ‘film’ that we observed comprised the remains of a fruit that had been treated with ammonia and was subsequently dried. The more delicate flower parts may have disintegrated upon drying.

It is unlikely that Kores had adequate flowering material at his disposal, otherwise J.J. Smith, who was a meticulous observer, would surely have used it to describe this species. We consider it plausible that Kores found some floral remains on a fruit and perhaps also examined a flower bud and prepared and illustrated a reconstruction. In the process, he could have been misled by the unusual characters of the species that we assume to be *S. gibbsiae*. These unusual characters are the following:

1. The lip callus is free from the lip surface and is raised and pressed against the ventral side of the column, making it seem as if the lip is ecallose.
2. The column has no appendage.
3. As the callus is pressed against the column (but not adnate to it), it resembles and appears to replace the appendage that many other species of *Stigmatodactylus* have in this position (which also suggests that this appendage has a function in those species that possess one).

An examination of the type material of *S. gibbsiae* in its current state did not enable us to reach an unequivocal conclusion as to many of its characters. The largest of the two remaining flower buds is only 3.8 mm long, and an attempt to dissect it would be destructive. There are three columns left on the fruits, but two of these are clearly damaged. One of the columns appears to be intact, and no appendage can be discerned on it. The shape of the labellar callus in our specimens agrees with that described by Kores [8], except that Kores illustrates this as lying on the lip surface along with an additional appendage on the column. In Kores’s illustration, the columnar appendage is unusually large and points obliquely upwards. The column with appendage thus produces almost the same lateral profile as the column with appressed callus in our specimens, and we believe that this is what Kores illustrated. The flower colour of our specimens, which were collected about 50 km W of the type locality, agrees with that recorded for *S. gibbsiae*. It is unclear where the detailed colour notes provided by Kores [8] come from (“outer perianth segments pale green, labellum brownish maroon”) because the annotation by Gibbs just states “flowers brown”. Kores also describes the colours of the vegetative parts, which are not mentioned at all by Gibbs, and which cannot be discerned from the uniformly brownish specimens.

Key to the species of *Stigmatodactylus* in New Guinea

**1a.** Lip callus consisting of a central crest flanked with several teeth … 2.

**1b.** Lip callus cymbiform or cup-shaped, not flanked with teeth, with or without two antennae … 3.

**Figure 4:**
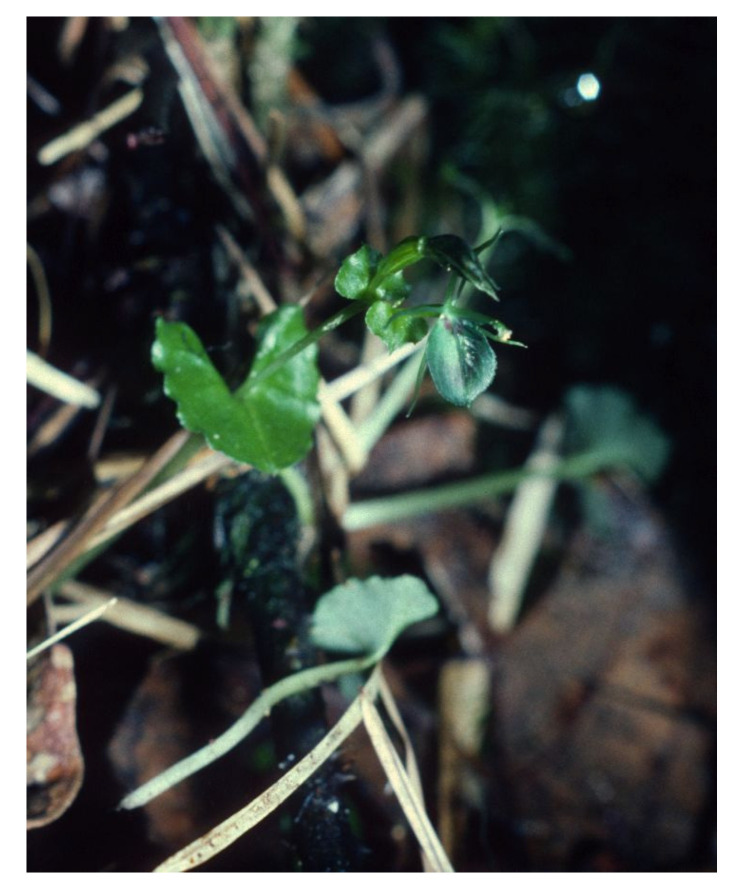
*Stigmatodactylus croftianus*, in situ, Mt. Kaindi, Papua New Guinea. *Schuiteman 17*. Photo: André Schuiteman.

**2a.** Plant with tuber. Lip broadly elliptical, bluish green with maroon spots at the base or pale green, 7–9-veined … *S. croftianus* (Kores) Kores [Figure 4].

**2b.** Plant without tuber. Lip obovate, dark purple with green markings, 15–17-veined … *S. variegatus* (Kores) Kores. Note: Key characters for the most part according to Kores [8]. The specimens illustrated as *S. variegatus* in Jones ([1], Figure 22.1) have the leaf characters of *S. croftianus* but lack a tuber and vary considerably in the details of the lip callus. They look intermediate between *S. variegatus* and S. *croftianus* in number of veins on the lip, suggesting that the two may not be distinct.

**3a.** Lip callus on either side with a long, subulate-filiform antenna, callus apex with two laterally flattened, triangular, vertical wings … *S. antennatus* Schuit., Saputra & Wanma.

**3b.** Lip callus without antennae, apex without wings … *S. gibbsiae* (Kores) Kores.

## 3. Materials and Methods 

Specimens were observed and photographed in the field; 3–4 samples were collected and preserved (dried and also stored in 70% ethanol). The samples were examined and photographed prior to preservation. Historic herbarium material was studied in the Natural History Museum (BM) in London. The fieldwork was carried out during 2023 and 2024 in the Arfak Mountains by a team of Universitas Papua (UNIPA), the West Papua Natural Resources Conservation Agency, and the Royal Botanic Gardens, Kew, for the purpose of documenting the orchid diversity of the Bird’s Head Peninsula of New Guinea. The second author (RS) also carried out fieldwork in the same area in 2025. Exact locality data are withheld in the interest of conservation.

## 4. Conclusions

More fieldwork around Anggi Gida Lake, where *Stigmatodactylus gibbsiae* was first collected and could still occur, is needed. Evidently, if a species was found there that agreed with Kores’s description and illustration, that is, if it had both a lip callus and a columnar appendage, our interpretation of *S. gibbsiae* would be incorrect, and the specimens we have identified as such belong to an undescribed species. For now, we consider it more likely that the discrepancies are due to a misinterpretation of poor type material of *S. gibbsiae*.

## Data Availability

All data are included in the text, except for precise collecting localities, which have been withheld in the interest of conservation.

## References

[1] Jones D., Pridgeon A.M., Cribb P.J., Chase M.W., Rasmussen F.N. (2001). *Stigmatodactylus*. Genera Orchidacearum Volume 2. Orchidoideae (Part 1).

[2] Robinson A.S. (2021). *Stigmatodactylus dalagangpalawanicum*[*sic*]: Orchidaceae. Curtis’s Bot. Mag..

[3] POWO (2022). Plants of the World Online. Facilitated by the Royal Botanic Gardens, Kew. https://powo.science.kew.org/.

[4] Kores P.J. (1995). A systematic study of the genus *Acianthus* (Orchidaceae: Diurideae). Allertonia.

[5] Clements M.A., Jones D.L. (2018). Notes on Australasian orchids 1: *Stigmatodactylus* and *Townsonia* (Diurideae). Aust. Orchid Rev..

[6] Lyon S. Molecular Phylogenetic Studies on Acianthinae.

[7] Smith J.J., Gibbs L.S. (1917). Orchidaceae. A Contribution to the Phytogeography and Flora of the Arfak Mountains.

[8] Kores P. (1991). A revision of the genus *Acianthus* (Orchidaceae) in Papuasia. Lindleyana.

[9] Kores P. (1992). New combinations in *Stigmatodactylus* (Orchidaceae). Novon.

[10] Gibbs L.S. (1917). A Contribution to the Phytogeography and Flora of the Arfak Mountains.

